# The three-dimensional structure prediction of human bitter taste receptor using the method of AlphaFold3

**DOI:** 10.1016/j.crfs.2025.101146

**Published:** 2025-07-14

**Authors:** Takafumi Shimizu, Rio Ohno, Michihiro Kayama, Kenta Aso, Yasuyuki Fujii, Yoshitomo Suhara, Vittorio Calabrese, Naomi Osakabe

**Affiliations:** a Systems Engineering and Science, Graduate School of Engineering and Science, Shibaura Institute of Technology, Japan; b Department of Bioscience and Engineering, Faculty of System Science and Engineering, Shibaura Institute of Technology, Japan; c Central Research Institute, ITO EN, Ltd., Japan; d SIT Research Laboratories, Shibaura Institute of Technology, Japan; e Department of Biomedical and Biotechnological Sciences, University of Catania, Italy

**Keywords:** Human bitter taste receptor, Alphafold3, Three-dimensional structure prediction, Extracellular loop, Transmembrane helix

## Abstract

Bitter taste receptors (T2Rs), a subfamily of G protein-coupled receptors, are expressed not only in oral tissues but also in extraoral sites, playing key roles in physiological processes such as the gut-brain axis. However, structural information on T2Rs is limited, with only two human T2Rs, T2R14 and T2R46, experimentally determined to date. This study explores the potential of AlphaFold3 (AF3), an advanced AI-based protein structure prediction tool, to predict the structures of 25 human T2Rs and compares them with those of the earlier AlphaFold2 (AF2). The accuracy of AF3 was evaluated by comparing the predicted structures of T2R14 and T2R46 with known experimental structures. Our results show that AF3 provides more accurate structural predictions than AF2 for these receptors, though the predicted local distance difference test scores for AF3 were unexpectedly lower across all T2R subtypes. Subsequent analysis indicated that significant structural variations were observed in the receptor's extracellular region, in contrast to a higher degree of structural consistency in the intracellular region. Clustering based on sequence identity and root mean square deviation highlighted distinct groupings among the receptors. The structural properties of these T2Rs may be related to their ability to recognize thousands of diverse bitter substances through interaction with the taste receptor-specific G protein, α-gustducin. The present study provides evidence that AF3 can advance our understanding of T2R structure and research into the biological activity of T2R-ligand interactions in health-related processes, including risk reduction of obesity and diabetes.

## Highlights

• Structural predictions of 25 human bitter taste receptors (T2Rs) were performed.
• AlphaFold3-predicted structures showed improved accuracy for T2Rs.
• Sequence and structural similarities among 25 T2Rs were analyzed.

## Graphical abstract

**Figure:**
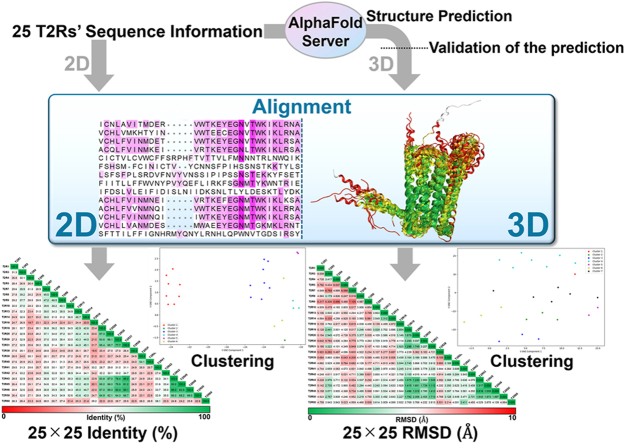

## Abbreviations used

T2R: bitter taste receptor
GPCR: G protein-coupled receptor
CNS: central nervous system
cryo-EM: cryo-electron microscopy
AF: AlphaFold
mmCIF: Macromolecular Crystallographic Information File
PDB: Protein Data Bank
MOE: Molecular Operating Environment
pLDDT: predicted local distance difference test
RMSD: root mean square deviation
t-SNE: t-distributed stochastic neighbor embedding
TM: transmembrane helix
ECL: extracellular loop

## 1 Introduction

The bitter taste sensation is mediated by bitter taste receptors (T2Rs), a subfamily of G protein-coupled receptors (GPCRs) expressed in taste receptor cells on the tongue (Andres-Barquin and Conte, 2004). The human genome was reported to contain 25 different T2R genes and 8 pseudogenes, predominantly located on chromosomes 7 and 12. T2Rs are known to be expressed throughout the body, not only in the taste-sensing oral tissues (Rozengurt et al., 2006; Tuzim and Korolczuk, 2021). These extraoral T2Rs have recently attracted attention for their involvement in homeostasis. In particular, T2R expressed on neuropod cells in the gastrointestinal tract has been the focus of significant biological interest due to its substantial role in mediating the gut-brain axis, like other taste receptors (Buchanan et al., 2022, Kaelberer et al., 2020). In the context of physiological function, these T2Rs has been suggested to play a dual role in the secretion of gastrointestinal hormones into the bloodstream and the release of neurotransmitters to afferent nerves through the activation of bitter substances. This process has been shown to enhance glucose tolerance whilst reducing suppressing appetite via the central nervous system (CNS) (Osakabe et al., 2024a; Osakabe et al., 2024b; Sternini and Rozengurt, 2025).

It is believed that there are thousands of bitter substances that activate T2R, and more than 2000 of them have been registered to date in BitterDB (https://bitterdb.agri.huji.ac.il/dbbitter.php) (accessed 2025-07-18), a comprehensive online database on bitter substances (Ziaikin et al., 2025). Nevertheless, understanding of the interactions between bitter substances and T2Rs is still limited. One reason is that while there is only one type of taste receptor for other tastes, such as sweet or umami, there are 25 types of T2R, and it is unclear how they are expressed within cells. Olfaction is comparable to bitterness in that both are chemical sensations involving multiple receptors. However, while only one type of olfactory receptor is expressed in each sensory neuron, it has been suggested that a single cell may express 4 to 11 types of T2Rs. Furthermore, it has been reported that T2Rs expressed in the same cell may influence each other, adding to the complexity of understanding the mechanism of bitter taste reception (Behrens et al., 2007; Bystrova and Kolesnikov, 2021). The second reason is that the three-dimensional structure information of T2R is limited. X-ray crystallography was the first powerful strategy used to solve the structures of GPCR. Still, crystallizing GPCRs is extremely difficult and time-consuming, with ligands often required to stabilize the domains during crystallization (Bertheleme et al., 2013; García-Nafría and Tate, 2021). Although single particle cryo-electron microscopy (cryo-EM) has grown rapidly in recent years and revolutionized structural biology, there is still a paucity of structural information for taste receptors with a unique G protein, α-gustducin. To date, the 3D structures of only two human T2R, T2R46 and T2R14, have been characterized by cryo-EM studies (Hu et al., 2024; Kim et al., 2024; Peri et al., 2024; Tao et al., 2024; Xu et al., 2022).

Elucidation of the interactions between bitter substances and bitter taste receptors is a significant undertaking that must be undertaken with utmost urgency to extend the healthy lifespan of humans. This is due to the potential efficacy of such knowledge in the prevention and treatment of obesity and diabetes. However, it is thought that it would take a considerable amount of time to determine the structures of the remaining 23 human T2Rs. On the other hand, it is thought that the elucidation of these structures may be facilitated using AI prediction technology, which has developed rapidly in recent years. AlphaFold3 (AF3), the latest version of the Nobel Prize-winning AlphaFold (Jumper et al., 2021), was released in beta version in May 2024 (Abramson et al., 2024). The architecture for structure prediction has been substantially updated from AlphaFold2 (AF2), and a diffusion model has been adopted to enable prediction at all atomic granularities. This study examined the potential of utilizing AF3 to analyze interactions between T2Rs and their ligands. We employed AF3 to predict the structures of 25 human T2Rs and compared them with structures predicted by AF2. Additionally, we verified the accuracy of AF3 predictions for T2R14 and T2R46, for which experimental structures are available. Furthermore, the present study investigated the identity between the sequences of 25 human T2Rs and the structures predicted by AF3 and compared the predicted structures themselves to identify structurally conserved and variable regions across the T2R family.

## 2 Materials and methods

### 2.1 Materials

The predicted structures of human T2Rs by AF2 were obtained from AlphaFold Protein Structure Database (https://alphafold.ebi.ac.uk/) (accessed 2025-07-18) in Macromolecular Crystallographic Information File (mmCIF) format. Structure prediction of all human T2Rs by AF3 were performed within AlphaFold Server (https://alphafoldserver.com/) (accessed 2025-07-18). Sequence information for each T2R were obtained from UniProt (https://www.uniprot.org/) (accessed 2025-07-18). The experimental structures of T2R14 and T2R46, whose structures are currently known, were obtained from the Protein Data Bank (PDB; https://www.rcsb.org/) (accessed 2025-07-18). Fifteen structures (PDB code: 8RQL; TAS2R14 receptor bound to flufenamic acid and gustducin, 8VY7; CryoEM structure of Gi-coupled TAS2R14 with cholesterol and an intracellular tastant, 8VY9; CryoEM structure of Ggust-coupled TAS2R14 with cholesterol and an intracellular tastant, 8XQL; Structure of human class T GPCR TAS2R14-miniGs/gust complex with Aristolochic acid A, 8XQN; Structure of human class T GPCR TAS2R14-DNGi complex with Aristolochic acid A, 8XQO; Structure of human class T GPCR TAS2R14-Gi complex with Aristolochic acid A, 8XQP; Structure of human class T GPCR TAS2R14-Gustducin complex with Aristolochic acid A, 8XQR; Structure 2 of human class T GPCR TAS2R14-miniGs/gust complex with Flufenamic acid, 8XQS; Structure of human class T GPCR TAS2R14-DNGi complex with Flufenamic acid, 8XQT; Structure of human class T GPCR TAS2R14-Gi complex, 8YKY; Structure of human class T GPCR TAS2R14-Ggustducin complex with agonist 28.1, 9IIW; A local Cryo-EM structure of Bitter taste receptor TAS2R14, 9IIX; A Cryo-EM structure of Bitter taste receptor TAS2R14 with Ggust, 9IJ9; A Cryo-EM structure of Bitter taste receptor TAS2R14 with Gi complex, 9IJA; A local Cryo-EM structure of Bitter taste receptor TAS2R14 with Gi complex) as experimental structures of T2R14 (Hu et al., 2024; Kim et al., 2024; Peri et al., 2024; Tao et al., 2024) and three structures (PDB code: 7XP4; T2R46 in apo state, 7XP5; T2R46 in ligand free state, 7XP6; T2R46 in active state) of T2R46 (Xu et al., 2022) were obtained from PDB in PDB exchange/mmCIF format. Structure visualization and comparison were performed using the Molecular Operating Environment (MOE; ver. 2022.02) (Molecular Operating Environment MOE, 2022). For clustering analysis, K-means clustering algorithm was used (Likas et al., 2003). The K-means algorithm was obtained from the open-source machine learning library, scikit-learn (https://scikit-learn.org/stable/) (accessed 2025-07-18), in Python. Computations were carried out in the Google Colab environment, using Python version 3.11.11.

### 2.2 Structure prediction of human T2Rs by AF3

Structure prediction of human T2Rs were performed by AF3 by inputting sequence information obtained from UniProt into AlphaFold Server. Predictions were made five times for each T2R, and the models with the highest predicted template modeling score (quality of the estimation) were selected. mmCIF files of the predicted structures were imported into MOE and visualized according to the predicted local distance difference test (pLDDT) values using the AF-Coloring program provided by the CCG SVL Exchange (https://svl.chemcomp.com/) (accessed 2025-07-18).

### 2.3 Validation of AF3's prediction accuracy

The prediction reliability of AF2 and AF3 was compared by the global pLDDT value. Furthermore, the accuracy of the predictions was confirmed by 3D alignment of the predicted structures by each AF with the currently known experimental structures of T2R14 (Hu et al., 2024; Kim et al., 2024; Peri et al., 2024; Tao et al., 2024) and T2R46 (Xu et al., 2022), respectively. In addition, we utilized AF3's capability to predict protein-protein complexes to model the T2R-G protein complex and performed 3D structural alignment with the experimentally determined cryo-EM structure.

### 2.4 Structure comparison of predicted human T2Rs

To clarify structural differences between T2R subtypes, amino acid sequences were aligned, and based on them, the predicted structures by AF3 were 3D aligned. In addition, structures were colored based on root mean square deviation (RMSD) values to reveal regions of structural diversity. RMSD values were also calculated for each transmembrane helix (TM) and loop region to assess local structural variations. Furthermore, the sequence identity and structural discrepancies (RMSD values) between each T2R subtype were calculated using MOE by aligning the sequences and superimposing the structures. To identify clusters of similar sequences and structures, clustering was performed using the K-means algorithm. Dimensionality reduction and visualization of the clusters were carried out using the t-distributed stochastic neighbor embedding (t-SNE) method (Van Der Maaten and Hinton, 2008).

## 3 Results

### 3.1 Comparison of human T2R structures predicted by AF2 and AF3

**Fig. 1:**
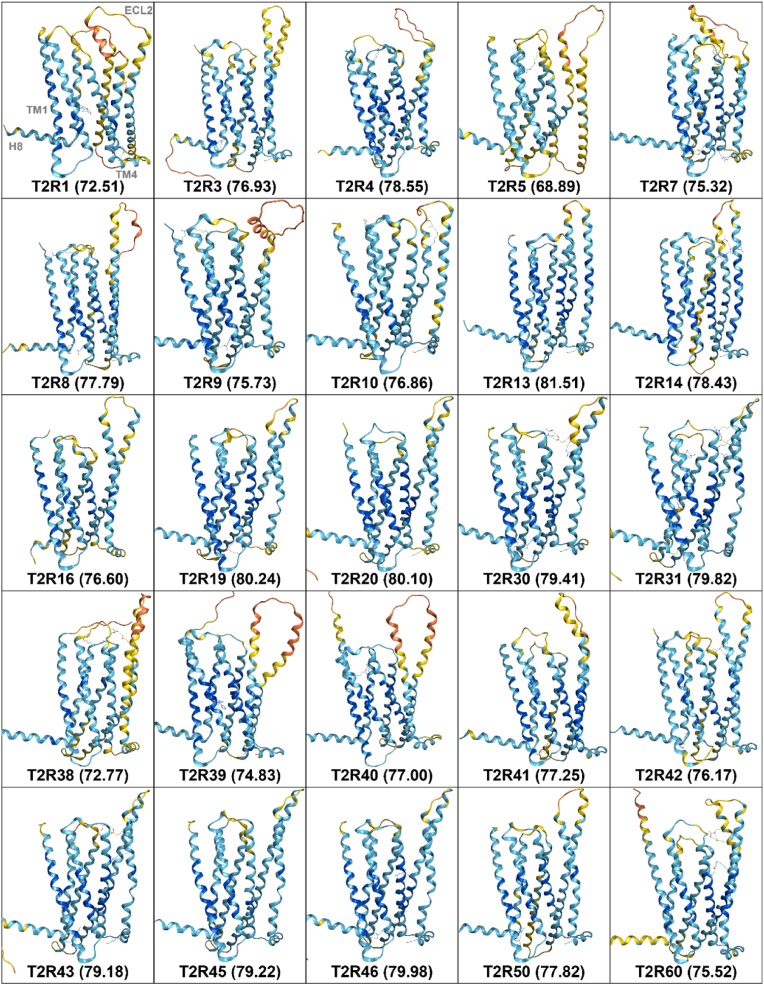
Predicted structures of all human T2R subtypes by AF3. For each T2R subtype, five prediction models were generated, and the best model was selected for further analysis. The global pLDDT values, which represent the confidence of the overall structural predictions, are indicated in parentheses for each T2R subtype. The pLDDT values ranged from 68.89 for T2R5 to 81.51 for T2R13. The structural models were colored with MOE based on pLDDT confidence levels: dark blue for pLDDT > 90 (very high), light blue for 90 > pLDDT > 70 (confident), yellow for 70 > pLDDT > 50 (low), and orange for 50 > pLDDT (very low).

**Fig. 2:**
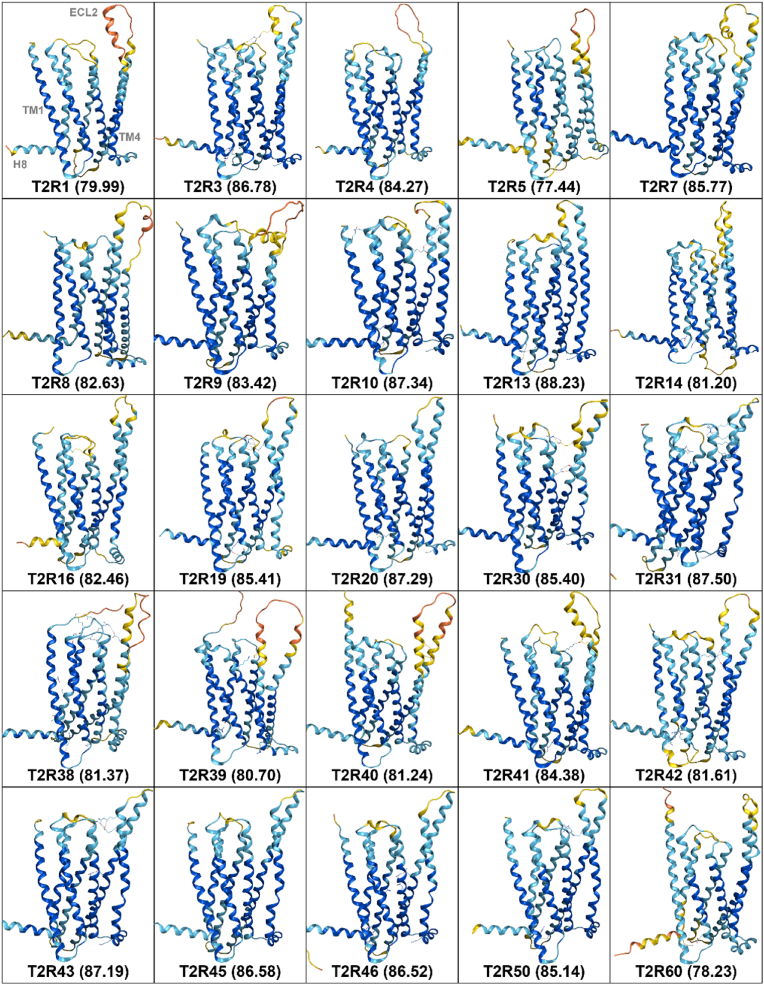
Predicted structures of all human T2R subtypes by AF2. The predicted structures of human T2Rs by AF2 were obtained from AlphaFold Protein Structure Database in mmCIF format. The pLDDT values ranged from 77.44 for T2R5 to 88.23 for T2R13. The structural models are colored with MOE based on pLDDT confidence levels: dark blue for pLDDT > 90 (very high), light blue for 90 > pLDDT > 70 (confident), yellow for 70 > pLDDT > 50 (low), and orange for 50 > pLDDT (very low).

**Fig. 3:**
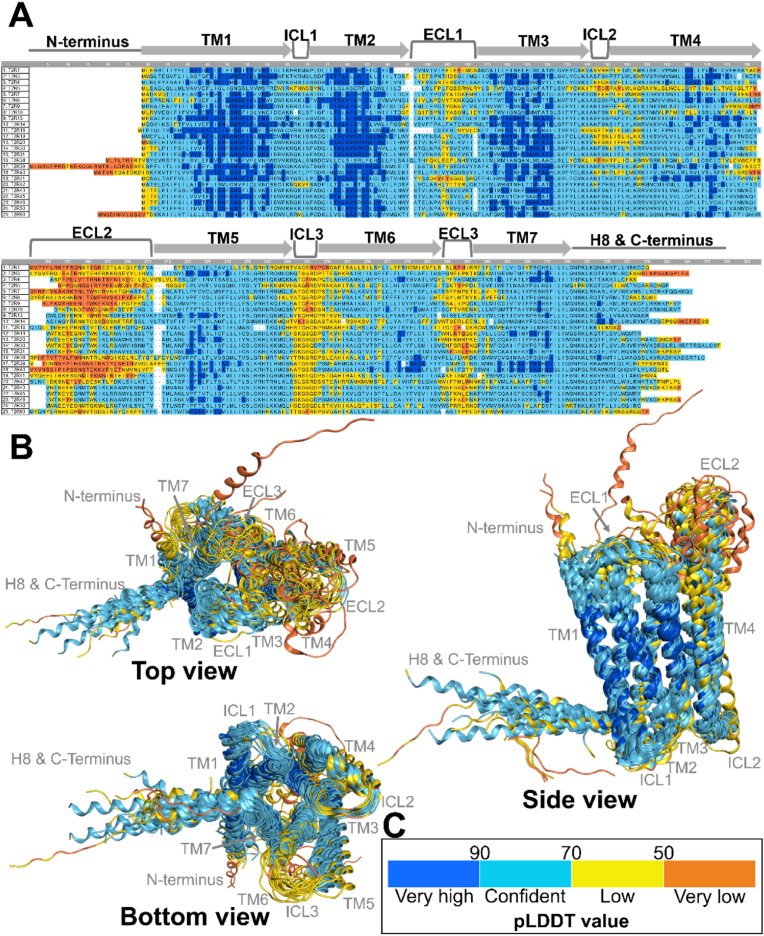
pLDDT values for each region of the T2Rs predicted by AF3. A, pLDDT values for each amino acid residue, along with the corresponding structure. The pLDDT values were higher in the TM regions, while lower values were observed in the ECL regions. Notably, higher pLDDT values were found in TM1, TM2, and TM3, whereas lower values were observed in ECL2. B, The 3D alignment of the predicted structures. In regions with high prediction accuracy, the structures showed high consistency, while in regions with low accuracy, considerable structural variation was observed. C, The pLDDT coloring criteria used in this study, where dark blue indicates pLDDT > 90 (very high), light blue indicates 90 > pLDDT >70 (confident), yellow indicates 70 > pLDDT > 50 (low), and orange indicates 50 > pLDDT (very low).

Fig. 1 presents the predicted structures of all human T2Rs generated by AF3. For each T2R subtype, predictions were performed five times, and the best model was selected for further analysis. The global pLDDT values, which indicate the confidence of the predictions, are shown in parentheses for each T2R. The pLDDT values ranged from a minimum of 68.89 for T2R5 to a maximum of 81.51 for T2R13. Contrary to our expectations, the global pLDDT values for each T2R were lower for all subtypes compared to AF2, which showed values ranging from 77.44 (T2R5) to 88.23 (T2R13) (Fig. 2). To clarify the regions with high and low prediction accuracy in T2R structures predicted by AF3, both the sequences and predicted structures were aligned in 2D and 3D, respectively. Fig. 3A shows the pLDDT values for each amino acid residue calculated by AF3, along with the corresponding structure. The pLDDT values were higher in the transmembrane helix (TM) regions, while lower values were observed in the extracellular loop (ECL) regions. Notably, higher pLDDT values were found in TM1, TM2, and TM3, whereas lower values were observed in ECL2. Fig. 3B illustrates the 3D alignment of the predicted structures. In regions with high prediction accuracy, the structures showed high consistency, while in regions with low accuracy, considerable structural variation was observed. Fig. 3C demonstrates the pLDDT coloring criteria used in this study, where dark blue indicates pLDDT > 90 (very high), light blue indicates 90 > pLDDT > 70 (confident), yellow indicates 70 > pLDDT > 50 (low), and orange indicates 50 > pLDDT (very low).

### 3.2 Comparison of the experimental structures of T2R14 and T2R46 with the predicted structure by AF3

**Fig. 4:**
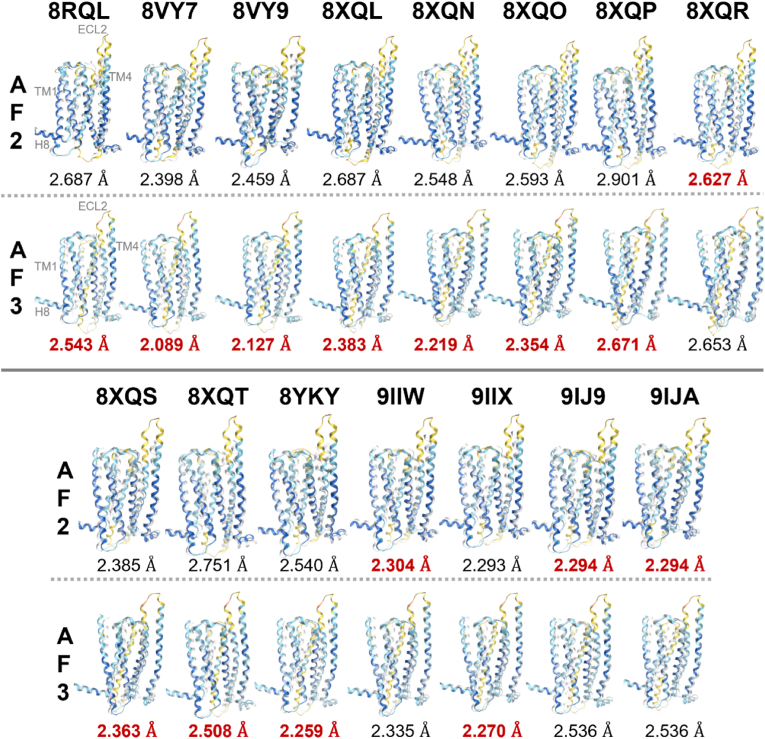
Alignment of the experimental structure of T2R14 with the predicted structures of AF2 and AF3. 8RQL: TAS2R14 receptor bound to flufenamic acid and gustducin; 8VY7: CryoEM structure of Gi-coupled TAS2R14 with cholesterol and an intracellular tastant; 8VY9: CryoEM structure of Ggust-coupled TAS2R14 with cholesterol and an intracellular tastant; 8XQL: Structure of human class T GPCR TAS2R14-miniGs/gust complex with Aristolochic acid A; 8XQN: Structure of human class T GPCR TAS2R14-DNGi complex with Aristolochic acid A; 8XQO: Structure of human class T GPCR TAS2R14-Gi complex with Aristolochic acid A; 8XQP: Structure of human class T GPCR TAS2R14-Gustducin complex with Aristolochic acid A; 8XQR: Structure 2 of human class T GPCR TAS2R14-miniGs/gust complex with Flufenamic acid; 8XQS: Structure of human class T GPCR TAS2R14-DNGi complex with Flufenamic acid; 8XQT: Structure of human class T GPCR TAS2R14-Gi complex; 8YKY: Structure of human class T GPCR TAS2R14-Ggustducin complex with agonist 28.1; 9IIW: A local Cryo-EM structure of Bitter taste receptor TAS2R14; 9IIX: A Cryo-EM structure of Bitter taste receptor TAS2R14 with Ggust; 9IJ9: A Cryo-EM structure of Bitter taste receptor TAS2R14 with Gi complex; 9IJA: A local Cryo-EM structure of Bitter taste receptor TAS2R14 with Gi complex. Transparent white ribbon structures represent experimental structures while structures colored by pLDDT values represent predicted structures by each AF, where dark blue indicates pLDDT > 90 (very high), light blue indicates 90 > pLDDT >70 (confident), yellow indicates 70 > pLDDT > 50 (low), and orange indicates 50 > pLDDT (very low). The smaller the RMSD value, the smaller the discrepancy between structures. RMSD values for AF structures with low RMSD values are shown in bold red. Among the 11 structures used (excluding 8XQR, 9IIW, 9IJ9, and 9IJA), AF3 demonstrated more accurate predictions compared to AF2. The highest resolution structure determined by cryo-EM (2.68 Å, 8VY7) showed the greatest similarity with the AF3 prediction, with an RMSD of 2.089 Å. The average RMSD for AF2 was 2.517 Å, while AF3 improved this to 2.390 Å.

**Fig. 5:**
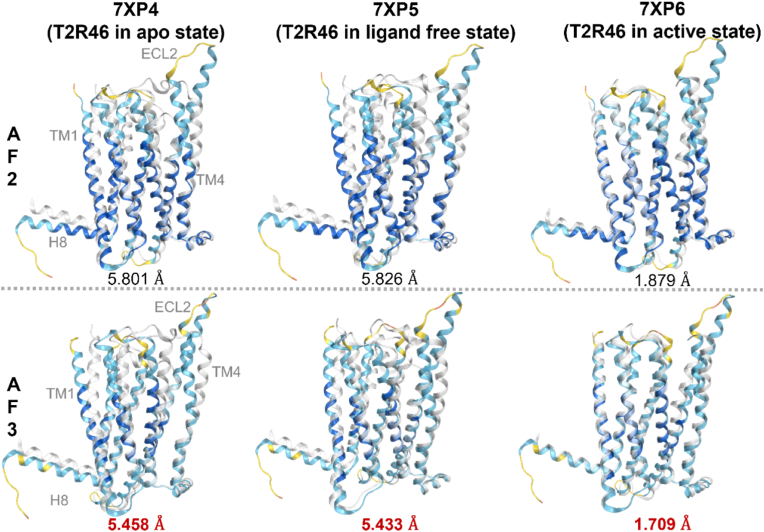
Alignment of the experimental structure of T2R46 with the predicted structures of AF2 and AF3. 7XP4: T2R46 in apo state; 7XP5: T2R46 in ligand free state; 7XP6: T2R46 in active state. Transparent white ribbon structures represent experimental structures, while structures colored by pLDDT values represent predicted structures by each AF, where dark blue indicates pLDDT > 90 (very high), light blue indicates 90 > pLDDT > 70 (confident), yellow indicates 70 > pLDDT > 50 (low), and orange indicates 50 > pLDDT (very low). The smaller the RMSD value, the smaller the discrepancy between structures. RMSD values for AF structures with low RMSD values are shown in bold red. AF3 provided more accurate predictions than AF2 for all three structures. The highest similarity was observed for the T2R46 in strychnine-bound active state, with an RMSD of 1.709 Å.

**Fig. 6:**
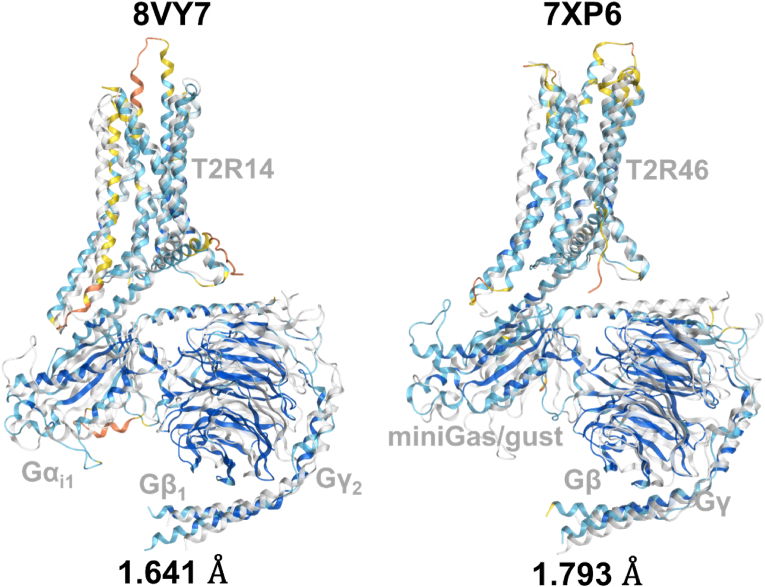
Predicted complex structures of T2R14 and T2R46 with G proteins using AlphaFold3. T2R14: PDB ID 8VY7, RMSD = 1.641 Å; T2R46: PDB ID 7XP6, RMSD = 1.793 Å. Transparent white ribbon structures represent experimental structures while structures colored by pLDDT values represent predicted structures by each AF, where dark blue indicates pLDDT > 90 (very high), light blue indicates 90 > pLDDT >70 (confident), yellow indicates 70 > pLDDT > 50 (low), and orange indicates 50 > pLDDT (very low). The smaller the RMSD value, the smaller the discrepancy between structures.

To evaluate the validity of the AF3 predictions, the predicted structures of T2R14 and T2R46 were superposed onto their experimentally determined structures, and the structural similarity was assessed by calculating RMSD values. For T2R14, the experimentally determined structures used were 8RQL (extracellular and intracellular flufenamic acid-bound structure), 8VY7 and 8VY9 (extracellular cholesterol and intracellular Cmpd. 28.1-bound structures), 8XQL, 8XQN, 8XQO, 8XQP (extracellular cholesterol and intracellular aristolochic acid A-bound structures), 8XQR (intracellular flufenamic acid-bound structure), 8XQS and 8XQT (extracellular cholesterol and intracellular flufenamic acid-bound structures), 8YKY (extracellular cholesterol and intracellular Cmpd. 28.1-bound structure), 9IIW and 9IIX (extracellular and intracellular Cmpd. 28.1-bound structures), 9IJ9 and 9IJA (extracellular cholesterol and intracellular Cmpd. 28.1-bound structures). Among the 11 structures used (excluding 8XQR, 9IIW, 9IJ9, and 9IJA), AF3 demonstrated more accurate predictions compared to AF2. The highest resolution structure determined by cryo-EM (2.68 Å, 8VY7) showed the greatest similarity with the AF3 prediction, with an RMSD of 2.089 Å (Fig. 4). The average RMSD for AF2 was 2.517 Å, while AF3 improved this to 2.390 Å. For T2R46, the experimentally determined structures included 7XP4 (apo state), 7XP5 (ligand-free state), and 7XP6 (strychnine-bound active state). AF3 provided more accurate predictions than AF2 for all three structures. The highest similarity was observed for the strychnine-bound T2R structure, with an RMSD of 1.709 Å (Fig. 5). In Figs. 4 and 5, the experimentally determined structures are depicted with transparent white ribbons, and the predicted structures are colored according to the pLDDT values using the same coloring criteria as described in Fig. 3C (Kim et al., 2024). Furthermore, AF3, which is capable of predicting protein–protein complexes, was employed to model the G protein-bound structures of T2R14 and T2R46. The predicted complex structures showed high structural similarity to the experimentally determined ones, with RMSD values of 1.641 Å for T2R14 (8VY7) and 1.793 Å for T2R46 (7XP6), respectively (Fig.6).

### 3.3 Similarities and differences between 25 predicted human T2R structures

**Fig. 7:**
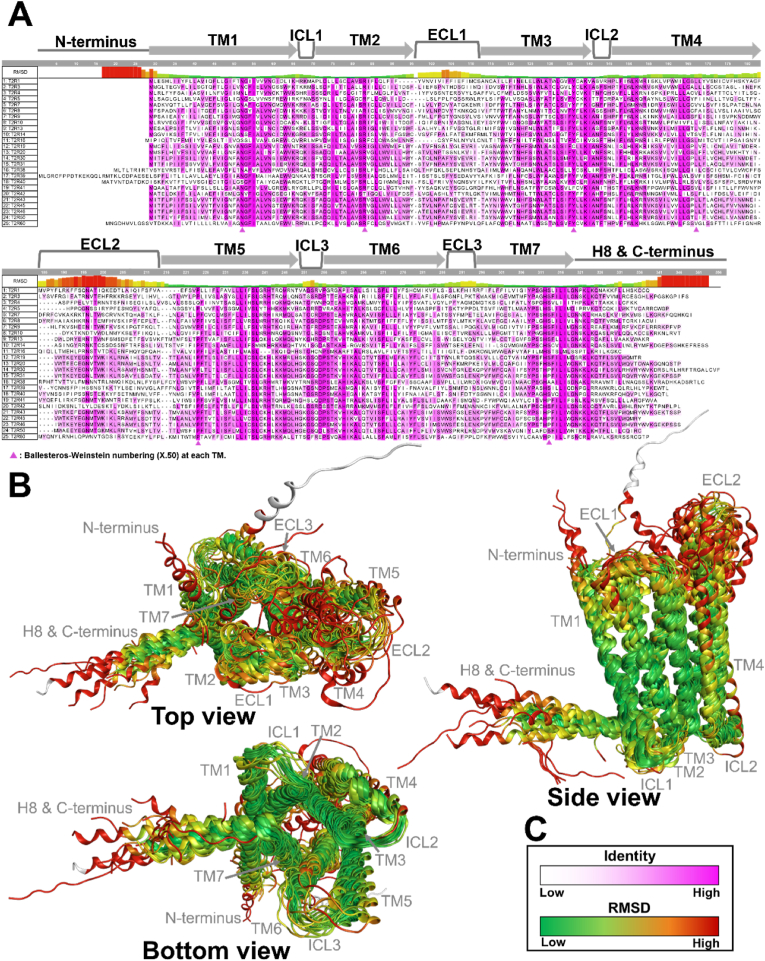
Similarities and differences between the amino acid sequences of 25 T2Rs and their predicted structures by AF3. A, the 2D alignment of amino acid sequences, the corresponding structures, and the structural variation of each residue, represented by RMSD values. Amino acid residues are color-coded based on sequence identity (%), with regions of higher identity shown in darker magenta. Additionally, the RMSD values for each residue, indicated above the sequence, transition from green to red as the values increase. The TM regions exhibited high sequence identity overall, while the extracellular regions showed lower identity, especially in the ECL structures where sequence diversity was observed. Furthermore, there was a tendency for sequence identity to decrease as it approaches the extracellular region, even within the TM domains. B, 3D alignment of the predicted structures. TM regions displayed high structural consistency, while the ECL structures, particularly ECL2, exhibited greater diversity. Additionally, the intracellular regions tended to show more consistent structures. C, The color-coding criteria used in the present figure, where higher identity percentages are represented by darker magenta, and RMSD values transition from green to red as the values increase.

**Fig. 8:**
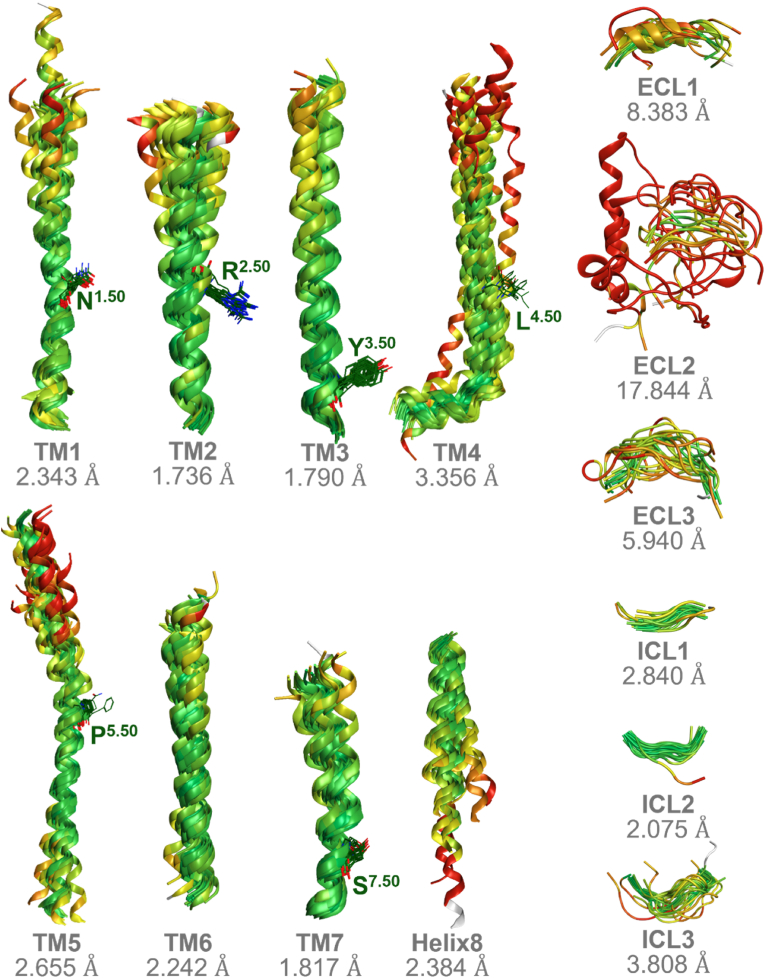
RMSD values for each helix and loop structure. Based on the full structural alignment shown in Fig. 7B, each helix (TM1–TM7, Helix 8) and loop region (ECLs and ICLs) was individually extracted, and RMSD values were calculated for each region. The RMSD values were as follows: TM1, 2.343 Å; TM2, 1.736 Å; TM3, 1.790 Å; TM4, 3.356 Å; TM5, 2.655 Å; TM6, 2.242 Å; TM7, 1.817 Å; Helix 8, 2.384 Å; ECL1, 8.383 Å; ECL2, 17.844 Å; ECL3, 5.940 Å; ICL1, 2.840 Å; ICL2, 2.075 Å; ICL3, 3.808 Å. RMSD values transition from green to red as the values increase.

**Fig. 9:**
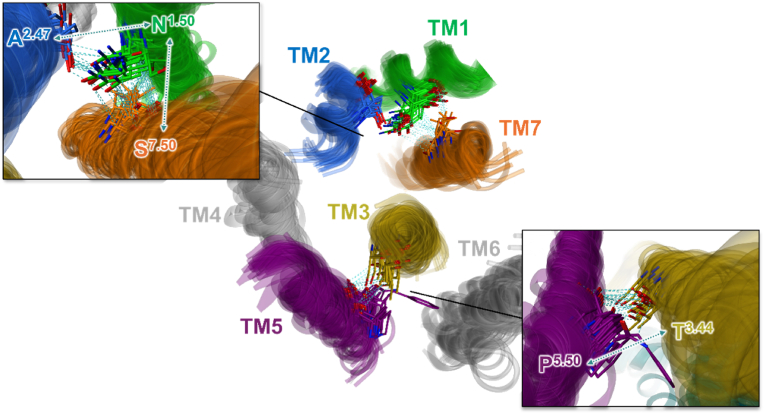
The role of conserved residues at the X.50 positions, based on the Ballesteros–Weinstein numbering system. Transmembrane helices are color-coded as follows: TM1 (green), TM2 (blue), TM3 (yellow), TM5 (purple), TM7 (orange), and TM4 and TM6 (gray). Hydrogen bonds between transmembrane helices were frequently observed across many T2Rs, particularly involving N^1.50^ and A^2.47^, as well as N^1.50^ and S^7.50^. Additionally, hydrogen bonding between P^5.50^ and T^3.44^ was commonly identified. The presence or absence of these interhelical hydrogen bonds for each T2R is summarized in Supplementary Table S1.

**Fig. 10:**
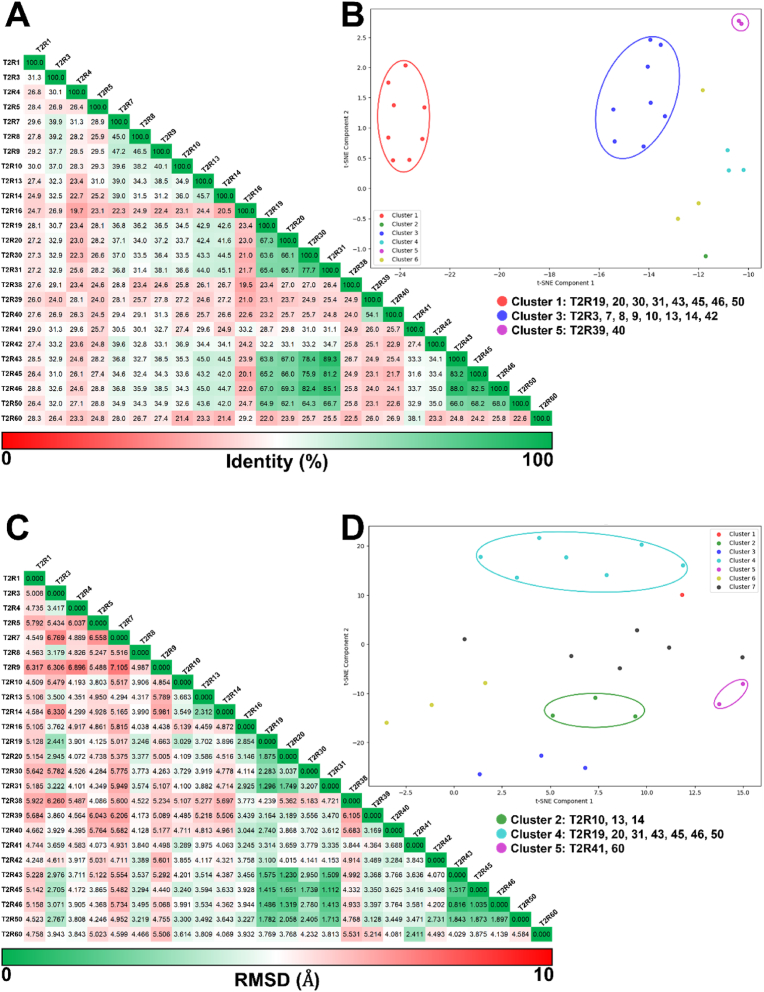
Clustering by sequence identity and RMSD value between each T2R subtype. A, Sequence identity among each T2R subtype. The sequence identity ranged from 19.5 % (between T2R16 and T2R38) to 89.3 % (between T2R31 and T2R43). B, Clustering results based on Fig. 10A. Circled clusters are clusters validated by silhouette analysis (Supplementary Fig. S2). T2R subtypes belonging to the validated cluster are shown at the bottom. C, RMSD values between each T2R subtype. The RMSD values varied from 0.816 Å (between T2R43 and T2R46) to 7.105 Å (between T2R7 and T2R9). D, clustering results based on Fig. 10C. Clusters circled as if they were clusters validated by silhouette analysis (Supplementary Fig. S3). T2R subtypes belonging to the validated cluster are shown at the bottom.

The results of the 2D alignment of T2R sequences and the 3D structural alignment based on these sequences, performed using MOE, are presented in Figs. 7 and 8. Fig. 7A displays the 2D alignment of amino acid sequences and their corresponding 3D structures. Amino acid residues are color-coded according to sequence identity (%), with regions of higher identity shown in darker magenta. In addition, RMSD values for each residue are shown above the sequences, with colors transitioning from green to red as the values increase. Overall, high sequence identity was observed in the TM regions, while both sequence identity and structural conservation tended to decrease toward the extracellular regions. Sequence diversity was particularly evident in the extracellular loop (ECL) regions (Fig. 7A). In terms of structural similarity, conserved structures were observed in the intracellular loop (ICL) and TM regions. Notably, TM2, TM3, and TM7 showed high structural conservation, with RMSD values of 1.736 Å, 1.790 Å, and 1.817 Å, respectively (Fig. 7, Fig. 8). In contrast, greater structural variability was observed in the ECLs, particularly in ECL2, which exhibited a significantly higher RMSD value of 17.844 Å (Fig. 7, Fig. 8). Fig. 7C illustrates the color-coding criteria used in Fig. 7, Fig. 8, where higher sequence identity is represented by darker magenta, and higher RMSD values are indicated by a color gradient from green to red. In addition, the role of conserved residues at the X.50 positions, based on the Ballesteros–Weinstein numbering system, was examined in each TM region to assess their contribution to structural stabilization. Hydrogen bonds between transmembrane helices were frequently observed in many T2Rs, particularly involving N^1.50^ and A^2.47^, as well as between N^1.50^ and S^7.50^. Furthermore, hydrogen bonding between P^5.50^ and T^3.44^ was also commonly identified (Fig. 9). The presence or absence of these interhelical hydrogen bonds for each T2R is summarized in Supplementary Table S1. The results of sequence identity (%) and RMSD values between each T2R subtype calculated by MOE are shown in Fig. 10A, C. The sequence identity of T2Rs ranged from 19.5 % (between T2R16 and T2R38) to 89.3 % (between T2R31 and T2R43), while the RMSD values varied from 0.816 Å (between T2R43 and T2R46) to 7.105 Å (between T2R7 and T2R9). Fig. 10B, D shows the results of K-means clustering based on this matrix. In this study, the number of clusters and the validity of the clustering were evaluated using silhouette analysis, and in Fig. 10B, D, the clusters confirmed to be valid are encircled, with the corresponding T2R subtypes indicated. As the result, three clusters were identified based on sequence identity: clusters consisting of T2R19, -20, -30, -31, -43, -45, -46, and -50; clusters consisting of T2R3, -7, -8, -9, -10, -13, -14, and -42; and clusters consisting of T2R39 and -40. Clustering based on RMSD values were validated for three clusters: clusters consisting of T2R19, -20, -31, -43, -45, -46, and -50; clusters consisting of T2R10, -13, and -14; and clusters consisting of T2R41 and -60. Detailed results of the K-means clustering are shown in Supplementary Figs. S1 and S2.

## 4 Discussion

The AF3 model was employed to predict the structures of 25 human T2Rs, and these predictions were then compared with the results obtained from AF2. The structure prediction for all T2R subtypes using AF3 yielded lower pLDDT values, an indicator of prediction reliability, across all subtypes compared to AF2, contrary to expectations (Fig. 1, Fig. 2). AF2 and AF3 have undergone significant architectural changes. AF3 employs a diffusion model that allows prediction at the atomic level of granularity (Abramson et al., 2024). Specifically, AF2 generates consistent pLDDT values for each residue, whereas AF3 calculates pLDDT values for all atoms that constitute a residue. These changes likely result in a lower overall pLDDT score in AF3 (Supplementary Fig. S3). Furthermore, the TM region showed higher pLDDT values than the ECL region (Fig. 3). The TM region, characterized by a high proportion of hydrophobic residues, might be attributed to a large amount of training data available from empirical studies and the ease of predicting arrangement patterns (Jacoby et al., 2006).

To assess the accuracy of the T2R structure predicted by AF3, this study compared it with the structure experimentally determined by cryo-EM (Fig. 4, Fig. 5, Fig. 6). When comparing the predicted structures to experimentally determined structures, AF3 demonstrated a greater capacity to predict conformations that closely align with the experimental data than AF2. This was particularly evident in the active state of T2R46, where AF3 provided highly accurate predictions for the ligand-binding state (Fig. 5).

Additionally, for the 15 experimentally determined T2R14 structures, which are also in ligand-binding states, AF3 achieved an average prediction accuracy below 2.5 Å, a commonly accepted benchmark for high-accuracy predictions (Fig. 4). A 2.5 Å RMSD corresponds to a global distance test total score of 80, which is the threshold for structural agreement with experimental data (Kryshtafovych et al., 2021).

Furthermore, the ability of AlphaFold3 to predict protein-protein complexes enabled high-accuracy modeling of T2R-G protein complexes (Fig. 6). This capability suggests significant potential for future studies involving large-scale molecular dynamics simulations that include both the receptor and its associated G protein, gustducin. Such simulations are expected to provide deeper insights into the structural dynamics of G protein activation in response to T2R activation. The enhanced predictive capability of AF3 compared to AF2 for protein conformations in the ligand-bound state renders it a potentially valuable instrument in biological research, including molecular docking and the prediction of ligand-receptor complexes, as well as in drug discovery. Nevertheless, the current implementation of the AlphaFold Server supports only a limited set of predefined ligands and does not allow users to incorporate external docking tools or custom ligand structures. As a result, its applicability to the investigation of bitter ligand–receptor interactions remains limited at this stage.

To investigate the structural diversity of human T2Rs, we calculated the RMSD values by superimposing the structures predicted by AF3. Overall, a high sequence identity was observed in the TM regions, particularly in TM2, TM3, and TM7, while both sequence and structural diversity were prominent in the loop structures (Fig. 8). Furthermore, this study revealed a tendency for sequence identity to decrease as it approaches the extracellular region, even within the TM domains. Additionally, the ICLs were found to connect the TM helices more shortly compared to the ECLs, resulting in an “inverted truncated cone” structure for the T2Rs. This structure, along with the pocket size and the structural diversity of the extracellular region, is thought to enable the receptor to accommodate a variety of ligands (Fig. 7, Fig. 8). Based on the consistent structures observed in the intracellular regions, we hypothesized that the unique G protein, α-gustducin, similarly binds to T2Rs in the intracellular regions. This suggests that a similar conformational change occurs during the dissociation of α-gustducin in response to T2R activation, providing important insights into the conditions required for activation. Furthermore, this study suggests that highly conserved amino acid residues such as N^1.50^, P^5.50^, and R^7.50^ contribute to the stabilization of the tertiary structure by forming interhelical hydrogen bond networks (Fig. 9). On the other hand, this study was not able to determine which specific structural features in the extracellular region contribute to ligand selectivity, nor could it confirm the presence or absence of the intracellular binding site previously reported for T2R14 (Hu et al., 2024; Kim et al., 2024; Tao et al., 2024). To address these questions, further integrative studies involving site-directed mutagenesis followed by *in vitro* assays, computational molecular dynamics simulations, and structural analyses such as cryo-EM are required. In addition to the overall trends in T2R, sequence identity (%) and RMSD values were individually calculated for each T2R subtype to clarify the sequence and structural similarities. The consistency of these similarities was further highlighted through K-means clustering. The results suggested that T2R19, -20, -31, -43, -45, -46, and -50 may be highly similar in sequence and structure (Fig. 10). All these TAS2R genes are located on chromosome 12 in a ∼400 kb region and have been shown to be closely related proteins in previous phylogenetic trees (Grau-Bové et al., 2022; Roudnitzky et al., 2015). This suggests that they may play similar roles in organisms. On the other hand, previous study has classified T2R20 and T2R50 as specialists, whereas T2R46 has been regarded as a generalist (Trius-Soler and Moreno, 2024). One possible explanation for this discrepancy is the research bias toward a subset of T2Rs that are more frequently studied. T2Rs are often investigated in the context of drug discovery, and receptors with restricted tissue expression patterns may be less commonly selected as research targets. In addition, the availability of a cryo-EM structure for T2R46 has undoubtedly facilitated its frequent use in structural and pharmacological studies (Xu et al., 2022). The expansion of *in silico* structural biology approaches, as demonstrated in the present study, may contribute to a more comprehensive and accurate assessment of ligand recognition profiles across the T2R family. Additionally, T2R10, -13, and -14 were grouped into the same cluster based on sequence identity and RMSD-based clustering. This similarity in sequence and structure may be reflected in their ligand recognition styles. While this study suggests that structural diversity in the extracellular region may enable responses to various ligands, it has also been reported that T2R14 possesses ligand-binding sites not only extracellularly but also intracellularly (Hu et al., 2024; Kim et al., 2024; Peri et al., 2024; Tao et al., 2024). These three T2Rs, which demonstrated sequence and structural similarity, may enable the recognition of ligands with different physicochemical properties by possessing intracellular ligand-binding sites as well.

However, one limitation of the present study is that a consistent agreement between sequence identity and structural similarity was not observed across other T2Rs. This discrepancy is likely attributable to the lower prediction accuracy of AF3 in flexible regions such as ECL2 (Fig. 7, Fig. 8). Notably, ECL2 is known to undergo substantial conformational changes upon receptor activation (Xu et al., 2022), underscoring the need for complementary approaches, such as cryo-EM-based structural elucidation and molecular dynamics simulations, to investigate time-dependent structural transitions. Additionally, clustering based on amino acid sequences did not reproduce the exact groupings reported in previous studies based on mRNA sequences (Trius-Soler and Moreno, 2024). This inconsistency may be due to codon degeneracy as well as differences in the clustering algorithms employed. In particular, the present study limited the number of clusters to increase the reliability of each group, which may have restricted the resolution of classification. More broadly, when considering T2Rs in a physiological context, it is important to acknowledge the possibility of cross-talk between T2Rs and other co-expressed receptors within the same cell (Behrens et al., 2007; Lee et al., 2014). Furthermore, individual variability in bitter taste perception, such as that associated with well-known polymorphisms in T2R38 (Trius-Soler et al., 2022), remains incompletely understood. Therefore, integrative research combining *in silico* structural biology, *in vitro* functional assays, and *in vivo* studies to elucidate T2R-mediated physiological effects will be essential for a more comprehensive understanding of T2R function.

## 5 Conclusion

This study demonstrated the potential of utilizing AF3 for analyzing interactions of T2Rs and their ligands by predicting the structures of 25 human T2Rs. The comparison between AF3 and AF2 predictions, alongside the verification of AF3 predictions for T2R14 and T2R46 using available experimental structures, highlighted the improved accuracy of AF3. Additionally, the investigation into the identity between the sequences of these receptors and their predicted structures provided further insights into their structural properties. Although AF3 excels at predicting ligand-binding conformations, further studies—such as molecular dynamics simulations and cryo-EM analysis—are required to fully understand ligand recognition, especially within intracellular regions of T2Rs.

## CRediT authorship contribution statement

**Takafumi Shimizu:** Data curation, Investigation, Methodology, Visualization, Writing – original draft. **Rio Ohno:** Data curation, Investigation. **Michihiro Kayama:** Data curation. **Kenta Aso:** Data curation. **Yasuyuki Fujii:** Formal analysis. **Yoshitomo Suhara:** Formal analysis. **Vittorio Calabrese:** Writing – review & editing. **Naomi Osakabe:** Conceptualization, Funding acquisition, Project administration, Supervision, Writing – review & editing, All authors discussed the results and commented on the manuscript.

## Declaration of competing interest

The authors declare that they have no known competing financial interests or personal relationships that could have appeared to influence the work reported in this paper.

## Data Availability

No data was used for the research described in the article.
