# Supplementary material for: The three-dimensional structure prediction of human bitter taste receptor using the method of AlphaFold3
Source: Curr Res Food Sci. 2025 Jul 14;11:101146. doi: 10.1016/j.crfs.2025.101146 (PMC12305321; doi:10.1016/j.crfs.2025.101146)
Supplement: Multimedia component 1 [file mmc1.docx]

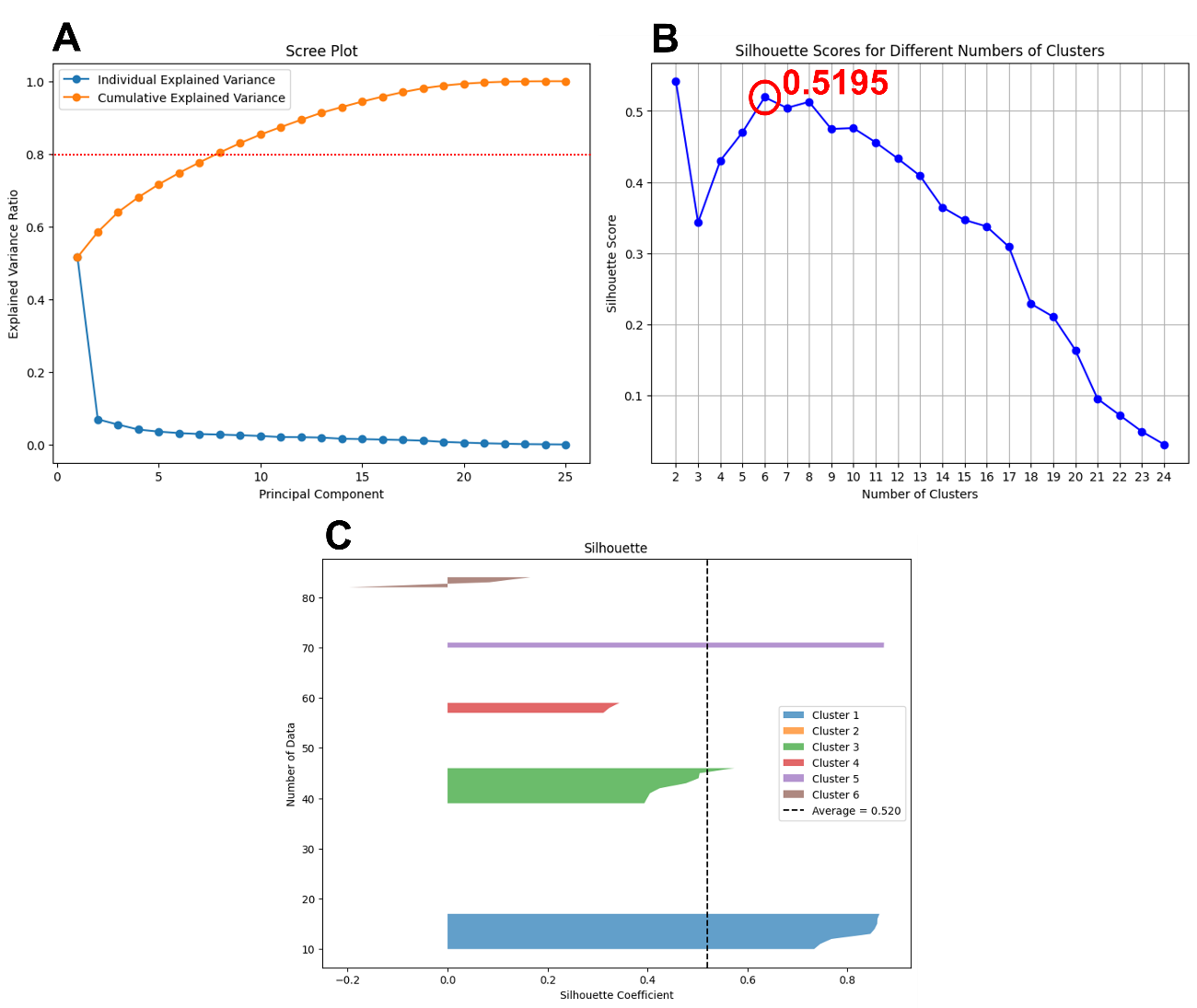
 **Supplementary Figure S1. Detailed results of K-means clustering based on sequence identity.** A, Scree plot analysis. In this study, clustering was performed using the first 8 principal components, which accounted for a cumulative explained variance of over 0.8. B, Silhouette score analysis for different numbers of clusters. A silhouette score of 0.5195 was obtained when the number of clusters was set to 6. C, Silhouette analysis for evaluating the validity of each cluster. The validity of clusters 1, 3, and 5 was confirmed.


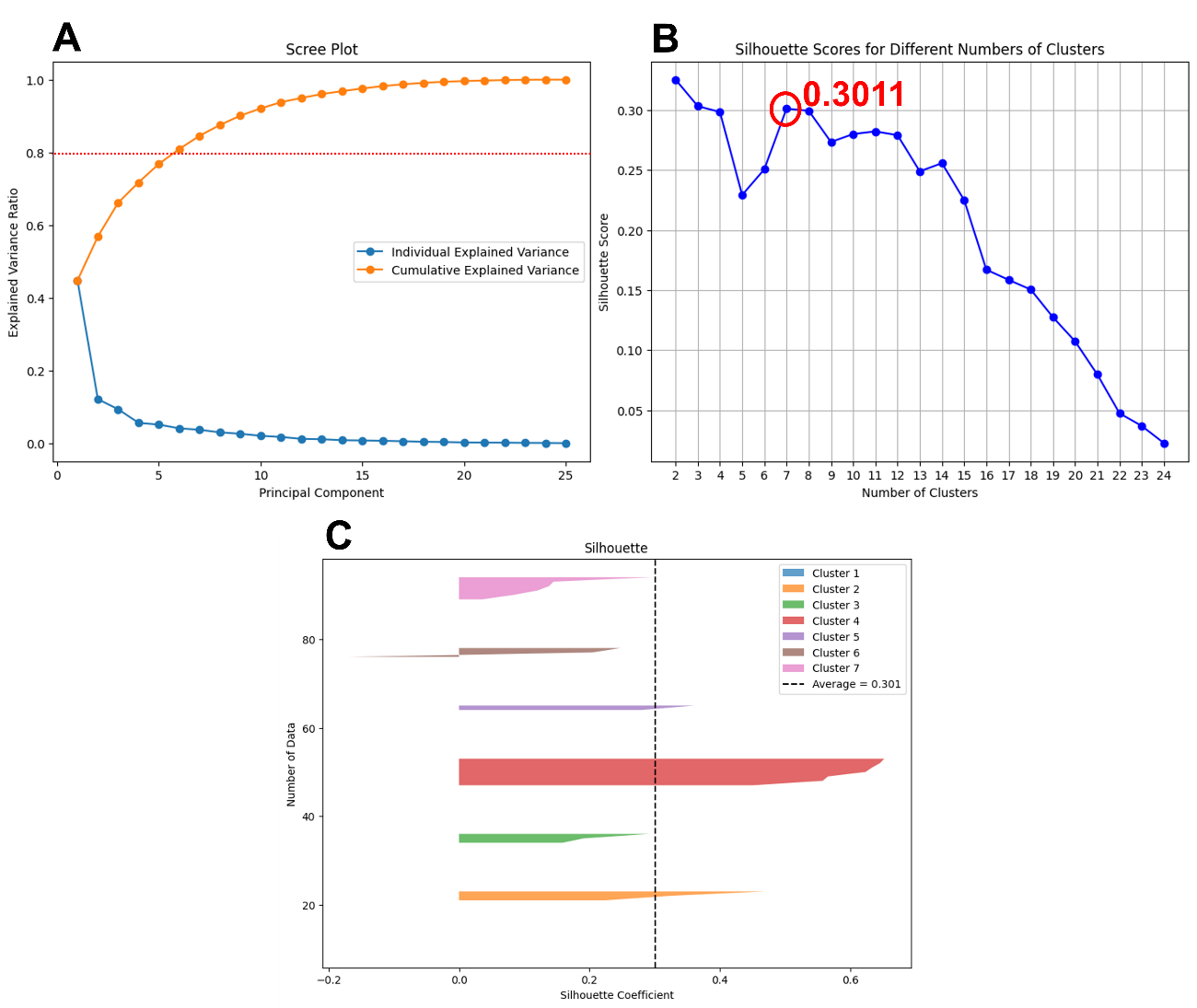


**Supplementary Figure S2. Detailed results of K-means clustering based on RMSD.**

A, Scree plot analysis. In this study, clustering was performed using the first 6 principal components, which accounted for a cumulative explained variance of over 0.8. B, Silhouette score analysis for different numbers of clusters. A silhouette score of 0.3011 was obtained when the number of clusters was set to 7. C, Silhouette analysis for evaluating the validity of each cluster. The validity of clusters 2, 4, and 5 was confirmed.


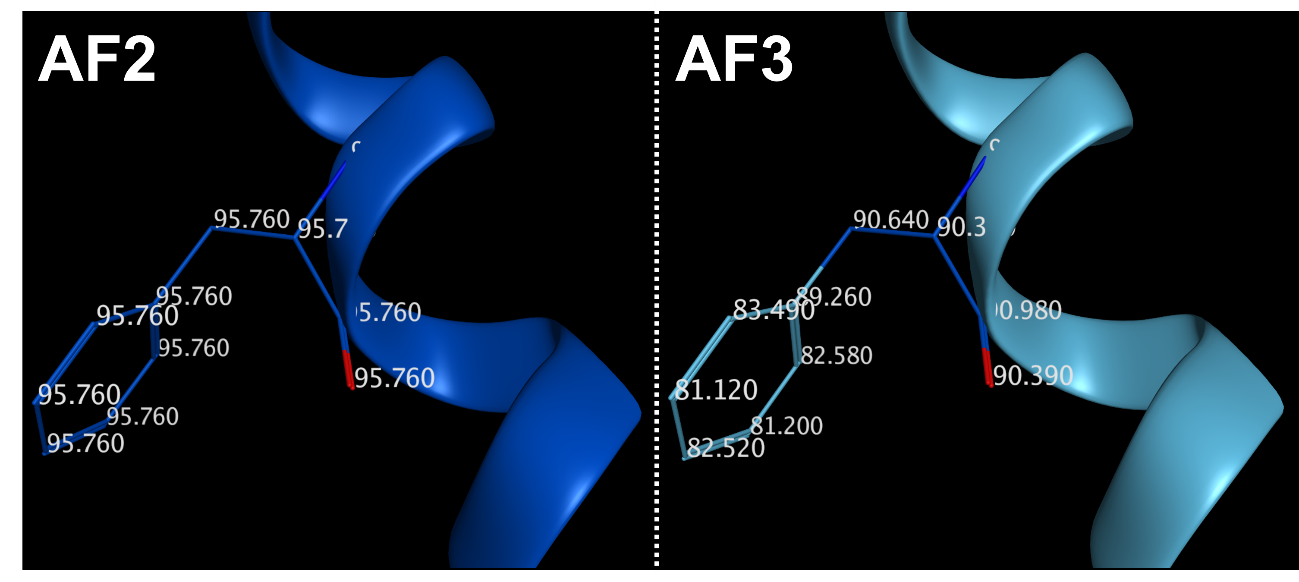


**Supplementary Figure S3. Assignment of pLDDT values to amino acid residues by AF2 and AF3.** In AF2, pLDDT values are assigned uniquely to each residue, providing specific confidence scores for each. In contrast, AF3, utilizing a diffusion model, outputs pLDDT values at an all-atom granularity, with residue-level pLDDT being the average of these atomic values. Dark blue indicates pLDDT > 90 (very high), light blue indicates 90 > pLDDT > 70 (confident).

**Supplementary Table S1. Summary of interhelical hydrogen bonds involving conserved residues in human T2Rs.** The presence or absence of interhelical hydrogen bonds involving conserved residues at Ballesteros–Weinstein positions (N^1.50^, A^2.47^, S^7.50^, P^5.50^, and T^3.44^) across 25 predicted human T2R structures.

|  | N^1.50^-A^2.47^ | N^1.50^-S^7.50^ | P^5.50^-T^3.44^ |
| --- | --- | --- | --- |
| T2R1 | **-** | **○** | **○** |
| T2R3 | **○** | **-** | **-** |
| T2R4 | **○** | **○** | **○** |
| T2R5 | **-** | **-** | **○** |
| T2R7 | **○** | **○** | **○** |
| T2R8 | **○** | **-** | **○** |
| T2R9 | **-** | **○** | **○** |
| T2R10 | **○** | **○** | **○** |
| T2R13 | **○** | **○** | **○** |
| T2R14 | **-** | **○** | **-** |
| T2R16 | **-** | **○** | **○** |
| T2R19 | **○** | **-** | **-** |
| T2R20 | **○** | **○** | **○** |
| T2R30 | **○** | **○** | **○** |
| T2R31 | **-** | **○** | **○** |
| T2R38 | **-** | **○** | **○** |
| T2R39 | **○** | **○** | **○** |
| T2R40 | **○** | **○** | **○** |
| T2R41 | **-** | **○** | **-** |
| T2R42 | **-** | **○** | **○** |
| T2R43 | **-** | **○** | **○** |
| T2R45 | **-** | **○** | **○** |
| T2R46 | **-** | **○** | **○** |
| T2R50 | **○** | **○** | **○** |
| T2R60 | **-** | **○** | **○** |
